# Health-related factors and dysregulation of epigenetic related genes in metabolic syndrome trigger finger patients and smoker trigger finger patients: preliminary analysis of patient-derived sample

**DOI:** 10.1186/s13018-023-04271-w

**Published:** 2023-10-18

**Authors:** Abdulaziz M. F. Shayea, Amna A. Alshatti, Danah H. Alfadhli, Almutairi Fatimah Ibrahim, Mariam Kh. Almutairi, Mohammed Sh. Nadar

**Affiliations:** 1 Occupational Therapy Department, Faculty of Allied Health Science, Kuwait University, P.O. Box 24923, 13110 Safat, Kuwait; 2 Anatomy Msc. in Neuroscience Felid, Departments of Anatomy, Faculty of Medicine, Kuwait University, P.O. Box 24923, 13110 Safat, Kuwait; 3 Departments of Molecular Biology, Faculty of Graduate Studies, Kuwait University, P.O. Box 24923, 13110 Safat, Kuwait

**Keywords:** Trigger fingers, Metabolic syndrome, Smoking, inflammation

## Abstract

**Purpose:**

To investigate the health-related factors and analyze the expression of epigenetic related genes and inflammatory genes in metabolic syndrome Trigger Finger (TF) and smoker TF.

**Methods:**

Samples from patients’ fingers with symptomatic TF were collected. There were seven groups: healthy control group, carpal tunnel syndrome (as a control for gene expression analysis), TF, diabetic TF, hypertensive TF, dyslipidemic TF and smoker TF. The expression levels of epigenetic related genes and inflammatory genes in metabolic syndrome TF and smoker TF were evaluated by the reverse transcription–polymerase chain reaction (RT-PCR) technique. The Perceived Stress Scale (PSS), Pittsburgh Sleep Quality Index (PSQI) questionnaires, disability of the arm, shoulder and hand (DASH) and numeric pain rating scale were given to the participants to fill out.

**Results:**

There was a significant increase in hand dysfunction in the metabolic TF groups and smoker group compared to the TF group (*p* < 0.0001). The stress levels of the smoker TF group and TF with hypertension group were significantly increased compared with those in the TF group (*p* < 0.03) and (*p* < 0.021), respectively. On the other hand, there was a significant increase in the COL-I, COL-II and TNF-α gene expression of the metabolic TF groups and smoker group (*p* < 0.0001).

**Conclusions:**

Health-related factors in the TF tendons was highly associated with the level of inflammation and genetic alteration in TF metabolic syndromes and smoker TF patients. Therefore, further investigation is required to examine the combination of occupational therapy, gene expression, and health-related factors as a promising method of managing TF.

## Introduction

Hand functions could be affected by several causes, such as congenital or acquired conditions, and traumatic injuries, which lead to significant stiffness and loss of function [1]. The clicking sound produced when an affected finger is extended and flexed gives the disease its common name, trigger finger [2]. Inflammation and hypertrophy of the retinacular sheath are the two main contributing factors to trigger finger, also known as stenosing tenosynovitis. It is a common musculoskeletal illness that manifests as a number of unpleasant and unsettling symptoms [3]. This retinacular sheath forms a pulley system consisting of a series of cruciform pulleys and annular pulleys in each digit, aiming to maximize the flexor tendon’s force production and motion efficiency [4]. This causes significant functional impairment associated with remarkable loss of motion along with pain, clicking, and catching of the affected digit. The trigger finger's first annular pulley (A1), which is the proximal part of the tendon sheath, is by far the pulley that is most frequently damaged [5]. The creation of tendon friction is caused by a thickened A1 pulley and increasing deterioration of the inner fiber cartilaginous gliding surface. This causes nodular change and inflammation in the tendon [6]. Obesity, impaired fasting glucose, increased blood pressure, and dyslipidaemia are among the metabolic features that make up the metabolic syndrome [7].

According to a study, trigger finger occurs in 80% of people with metabolic syndrome [8]. Additionally, 11.50% of patients with metabolic syndrome did not react to local corticosteroid injection therapy, according to Rho et al. [9]. According to the authors, this would enough to demonstrate the necessity for additional research on the link between the metabolic syndrome and trigger finger [9]. Moreover, secondary trigger finger is well described in the literature, and was shown to be related to multiple conditions, such as rheumatoid arthritis and diabetes, as well as various tumors, neoplasm and other metabolic disorders [10]. Diabetes is a condition caused by cellular resistance to insulin. Patients with diabetes have been shown to have higher prevalence of TF compared to patients without diabetes, although some researchers have found no difference in TF occurrence between diabetic and non-diabetic patients [11, 12].

The surprising effects of nicotine on the hands increase the risk of developing trigger finger by producing chemicals that worsen the hand condition. One study suggests that smoking causes serious complications and poor bone healing. It also negatively affects surgical outcome. The results claim that, in trigger finger release, smoking was a factor of postoperative surgical site infection [13].

The underlying pathophysiological mechanism of TF has been studied, including recent interest in altered gene expression in these patients. There is evidence of the upregulation of collagen genes, downregulation of the extra-cellular matrix, proteolytic enzymes, and dysregulation signaling pathways including cytokines such as TGF-b1 and PI3K [14]. When released by fat cells, certain cytokines, such as tumor necrosis factor alpha (TNF-α) and interleukin-6 (IL-6), cause an excess of other pro-inflammatory cytokines [15]. The biggest positive fold change was displayed by collagen types 1a1, 3a1, and 5a2, as well as by aggrecan, biglycan, COL1A2, COL11A1, COL5A1, and COL2A1, and downregulation of MMp-3 and TIMP3 in the trigger finger tendons [16].

## Objectives

The purpose of the study was to investigate and differentiate the health-related factors associated with smokers’ TF and metabolic syndromes TF, and to analyze and differentiate the related epigenetic (COL-I and COL-II) genes and inflammatory (TNF-α) gene among all groups.

## Hypothesis

We hypothesize that there are differences in health-related factors and gene expression among metabolic trigger finger patients and smoker trigger finger patients.

## Material and methods

### Study design and instruments

The Perceived Stress Scale (PSS-10), Pittsburgh Sleep Quality Index (PSQI), Numerical Rating Scale (NRS), Disability of Arm, Shoulder and Hand Questionnaire (DASH), and Satisfaction with Life Scale (SWLS) were the standardized end measures used in this study. These evaluations served as validated outcome measures. These assessments were used by occupational therapist to evaluate specific health-related factors. These standardized assessments were used consistency for all groups prior one week of the operation appointment. The Pittsburgh Sleep Quality Index (PSQI) assesses the quality of sleep while the Perceived Stress Scale (PSS-10) assesses stress experienced over the previous month. The pain was evaluated last month using the Numerical Rating Scale (NRS). Hand function is assessed using the Disability of Arm, Shoulder and Hand Questionnaire (DASH). Additionally, the SWLS (Satisfaction with Life Scale) tests for life satisfaction. They are all self-reported questionnaires that have been standardized [17–20]. The goal and administration of these assessments were described in further detail in administration of assessments section.

### Participants and patients’ samples

**Fig. 1:**
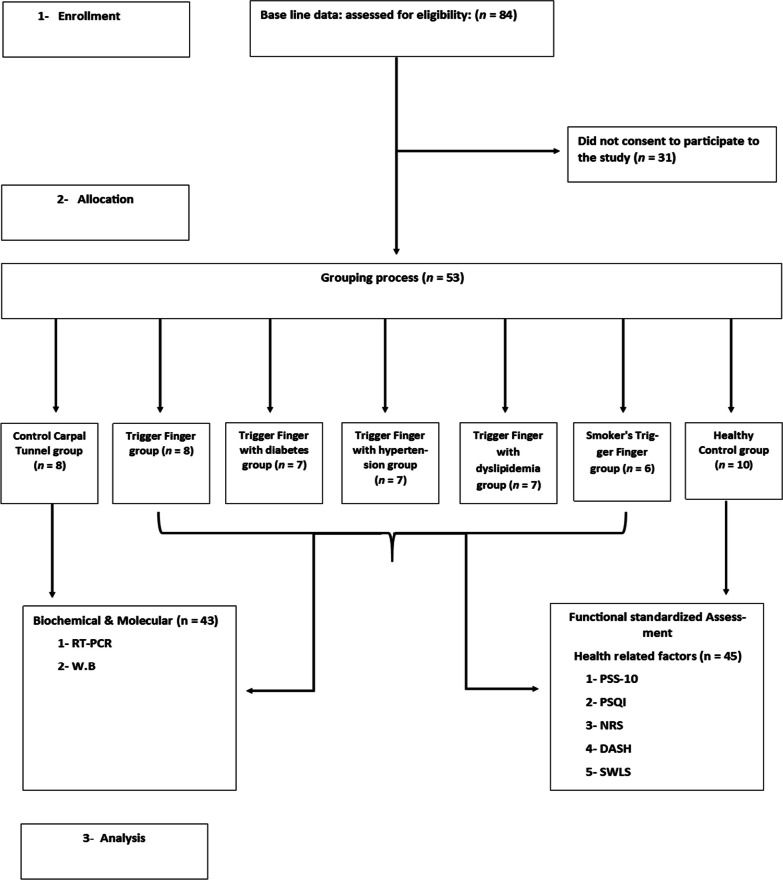
Study flow diagram

Samples from fingers of patients with symptomatic trigger fingers (TF) were collected in the department of orthopedics et al.-Razi hospital after undergoing surgery of the A1 pulley to release trigger finger. The patients who had tenderness at the A1 pulley were categorized as shown in Table 1. There were two control groups in this study. The first control group is the carpal tunnel syndrome (CTS) and the second control group is healthy control group. The samples were obtained from patients with carpal tunnel syndrome (CTS) as a first control group because of the limitations when obtaining samples from healthy subjects [21]. The CTS control group is used to compare the gene expression level and protein expression level with TF patients by using RT-PCR and western blot techniques as it shown in flow char (Fig. 1). We divided the metabolic syndrome group into three groups. These group were TF with diabetes, TF with hypertension and TF with dyslipidaemia. Also, we added a smoker's TF groups. Inclusion criteria were patients over 21 years of age who had been diagnosed with one metabolic syndrome disease, either diabetes, hypertension, or dyslipidaemia. Whereas, regarding the smokers group, participants should not be diagnosed with any type of metabolic syndrome disease and have been smoking for at least 5 years with any type of nicotine. Exclusion criteria are as follows: combined metabolic syndrome diseases or smoking with metabolic syndrome diseases. Samples of all groups were transported to the laboratory, and dissected specimens were frozen and stored at –80 °C.

**Table 1:** Tabulation of treatment to respective groups

Groups	Treatment
1	Control (carpal tunnel)
2	Control (healthy individuals)
3	TF
4	Diabetes + TF
5	Hypertension + TF
6	Dyslipidaemia + TF
7	Smoking + TF

On the other hand, the reason of taking healthy control group is to compare the health-related factors with TF patients by using the Perceived Stress Scale (PSS-10), Pittsburgh Sleep Quality Index (PSQI), Numerical Rating Scale (NRS), Disability of Arm, Shoulder and Hand Questionnaire (DASH), and Satisfaction with Life Scale (SWLS), which were the standardized end measures as it shown in the flow chart (Fig. 1).

#### Ethical approval

Ethical approval was obtained from Kuwait University Health Sciences Center Research Ethical Committee (protocol code 195; dated 3 October 2022).

#### Informed consent

Informed consent was obtained from all participants prior to data collection.

### Procedure

Participants were asked to fill in the assessments before the surgical procedure. Samples were taken from patients undergoing A1 pulley release due to TF et al.-Razi hospital by surgeons in the orthopedic department. After categorizing and separating the patient samples into groups, the samples were immediately transported to the laboratory for examination.

### Instruments

#### DASH assessment

The Disability of the Arm, Shoulder and Hand (DASH) assessment is a questionnaire that is used to evaluate the impact of upper-extremity disorders on a person’s ability to perform daily activities. The assessment consists of 30 items that cover different aspects of upper-extremity function, such as activities of daily living, work-related tasks, and sport and recreation. The DASH assessment also includes a section on symptoms such as pain and tingling. The questionnaire is self-administered and scored on a scale from 0 (no disability) to 100 (most severe disability) [18].

#### Numeric rating scale

The Numeric Rating Scale (NRS) is a simple and commonly used tool for measuring pain intensity on a scale from 0 to 10. A score of zero represents “no pain” and 10 represents the “most intense pain possible.” Patients are asked to rate their level of pain by choosing a number on the scale that best represents their pain intensity [17].

#### PSQI assessment

The Pittsburgh Sleep Quality Index (PSQI) is a self-administered questionnaire used to assess an individual’s sleep quality and patterns over a one-month period. The questionnaire consists of 19 items that assess different aspects of sleep, including duration, latency, disturbances of sleep. The PSQI produces a final score ranging from 0 to 21, with lower scores represent better quality of sleep [19].

#### PSS assessment

The Perceived Stress Scale (PSS) is a self-administered questionnaire used to assess an individual’s perception of stress in their life over the past month. The questionnaire consists of 10 items that measure the degree to which an individual perceives their life as unpredictable, uncontrollable, and overwhelming. The PSS produces a total score ranging from 0 to 40, with higher scores indicating a higher perceived level of stress [19].

#### SWLS assessment

The Satisfaction with Life Scale (SWLS) is a short assessment designed to assess global cognitive judgment of satisfaction with the respondent’s life. It consists of 5 items that reflect how the participants perceived their lives using 7 points Likert scale (1 = “strongly disagree,” 7 = “strongly agree”). The SWLS produces a total score ranging from 5 to 35, with higher scores representing higher satisfaction in life [20].

#### Real-Time PCR

Pre-designed and validated TaqMan® gene expression assays (Thermo Fisher Scientific, Waltham, MA, USA) were utilized in real-time PCR procedures to measure the relative gene expression, as described in (Table 2), with -actin serving as an endogenous reference. A 2*TaqMan universal master mix and cDNA template from Thermo Fisher Scientific, Waltham, Massachusetts, USA, are the components of the real-time PCR process. The ending amount of 20 L was finalized with nuclease-free water and the 20*TaqMan test. Following that, these mixes were made ready in a 96-well reaction bowl. The 7500 Sequence Detection System was then used to process these mixtures. We cycled the manufacturer-recommended parameters. A calibrator was created using the gene expression in the experimental groups and measured through the ***2***^−ΔΔCT^ equation.

**Table 2:** The genes obtained from applied biosystem

Genes
COL1A1
(collagen type I alpha 1 chain)
Forward sequence: GATTCCCTGGACCTAAAGGTGC
Reverse sequence:
AGCCTCTCCATCTTTGCCAGCA
COL2A1
(collagen type II alpha 1 chain)
Forward sequence:
CCTGGCAAAGATGGTGAGACAG
Reverse sequence:
CCTGGTTTTCCACCTTCACCTG
TNF- α
Forward sequence:
CTCTTCTGCCTGCTGCACTTTG
Reverse sequence:
ATGGGCTACAGGCTTGTCACTC

#### Western blot

The A1 pulleys tissues were quickly isolated. They were placed directly in liquid nitrogen. After that, these segments were stored at − 70 °C. Then, these frozen segments were mixed with a radioimmunoprecipitation assay buffer: RIPA: #sc-24948, Santa Cruz. A 10 μL PMSF solution, 10 μL sodium orthovanadate solution, and 10–20 μL protease inhibitor cocktail solution was merged per mL of 1X RIPA Lysis buffer. This was carried out before lysing cells to prepare and complete RIPA. A total of 3 mL of complete RIPA was required for one gram of tissue. The total protein of the homogenizing sample was determined using the BioTek protein estimation instrument. Twenty-four % Mini-PROTEAN TGX Precast Protein Gels were used to run the Western Blot. Bio-Rad precision plus protein kaleidoscope, #161–0375, was used as the ladder. A total of 50 μg was placed into each well and run under a 100 V for 50–75 min. The PVDF membrane (Bio-Rad, #sc-24948, Santa Cruz Biotechnology, Dallas, TX) was saturated in methanol, distilled water, and 1 × transfer buffer, for 10 min each. The transferred gel to the membrane was at 75 V (2 gels), and for 75 min blotting. The membrane blocking occurred in blocking solution for 1 h at room temperature on a shaker. Western blot was used to determine the expression levels of various proteins such as TNF-α after resolution of the lysate on SDS-PAGE (Bio-Rad, Santa Cruz Biotechnology, Dallas, TX).

### Statistical analysis

Data are shown as the mean ± standard error of the mean. The data were analyzed using one-way ANOVA analysis of variance with Bonferroni post hoc test and Pearson correlation. This was achieved using Prism 5.0 (Graph Pad Software, Inc., La Jolla, CA, USA). A *p*-value of < 0.05 was considered statistically significant differences. Potential confounders (stress, sleep quality, pain, hand function and life satisfaction) were adjusted as per a Directed Acyclic Graphs (DAG), based on preexisting knowledge about their relationships with hand function, life satisfaction and gene expression. Further analysis adjustments to the association between the DAG results, stress, sleep quality and pain of hand function and life satisfaction with gene expression [22].

## Results

Table 3 demonstrates participants’ demographic data. A total of 53 participants were included in this present study; twenty-four (45%) were female and twenty-nine (55%) were male, with an age range of 43 to 61 years old (mean = 52 ± 8.62). Average BMI of all patients was 25.1. ten (19%) were healthy control, 8 (15%) were carpal tunnel patients, 35 (53%) were trigger finger and trigger finger with metabolic syndrome disease, and 10 (13%) were smoker trigger finger.

**Table 3:** Participants’ demographics and health characteristics

Number of participants	53	
Age (mean ± SD in years)	52 ± 8.62	
Gender (male/female)	29/24	55/45
Dominance (right/left)	35/18	66/44
Healthy control	10	19
Carpal tunnel syndrome	8	15
TF	8	15
Metabolic syndrome diseases		
Diabetes mellitus	7	13
Hypertension	7	13
Dyslipidaemia	7	12
Smokers	6	13
Operated finger		
Thumb	10	28
Index	9	26
Middle	16	44
Ring	0	0
Little	0	0
Operated on dominant hand		
Yes	43	100
No	0	
Previous corticoid injections		
Yes		
Once	12	36
Twice	8	24
Three times	8	24
No	7	20

Thirty-five (66%) of the respondents were right-hand-dominant, eighteen (44%) were left-hand-dominant. Ten healthy control participants were included in this study. A total of 6 (60%) of the healthy control respondents were male, while 4 (40%) of them were female. Of the 43 patients that underwent operation, 8 participants (representing 15% of the respondents) had carpel tunnel. A total of 27 of the participants were trigger finger patients with metabolic syndrome diseases; 7 (representing 26% of the respondents) were diabetic trigger finger patients, 7 (representing 26% of the respondents) were hypertensive trigger finger patients, 7 (representing 26% of the respondents) were dyslipidemic trigger finger patients, and 6 (representing 22% of the respondents) were smoker trigger finger patients. The finger that was most frequently operated on, in 35 of the trigger finger patients (representing 44% of the respondents), was the middle finger. A total of 28 trigger finger participants had corticosteroid; 12 (representing 36% of the respondents) were injected once, 8 (24%) were injected twice and 8 (24%) were injected three times.

There was no significant difference between the baseline characteristics of participant groups, except for BMI/kg between healthy control and the five experimental groups *p* < 0.0001 (Table 4).

**Table 4:** Baseline characteristics of participants

Characteristic	Healthy Control Group (*n* = 10)	Experimental: Five Groups Total = 35	*p*-Value	Between metabolic syndrome TF groups and smoker's TF group Total = 35	*p*-Value
Mean (± SD) Age	53 ± 6.721	53 ± 8.612	0.4	53 ± 9.281	0.171
% Male	60%	54%	0.194	44%	0.265
Mean (± SD) BMI (Kg/m^2^)	21.4 ± 2.215	28.9 ± 3.321	0.0001 *	28.75 ± 2.312	0.173
% Dominant Hand					
Right	80%	77%	0.725	72%	0.906
Left	20%	23%		28%	
Mean (± SD) Operated Finger					
Thumb	–	28%	–	24%	0.601
Index		26%			
Middle		44%		76%	
Ring					
Little					
Previous corticosteroid injection					
No		20%	-	20%	0.605
Yes	–	80%		80%	
Once		34%		34%	
Twice		23%		23%	
Three times		23%		23%	
Previous Rehabilitation sessions					
No	–	52%	–	52%	0.935
Yes		48%		48%	

^*^P-value < 0.05

**Fig. 2:**
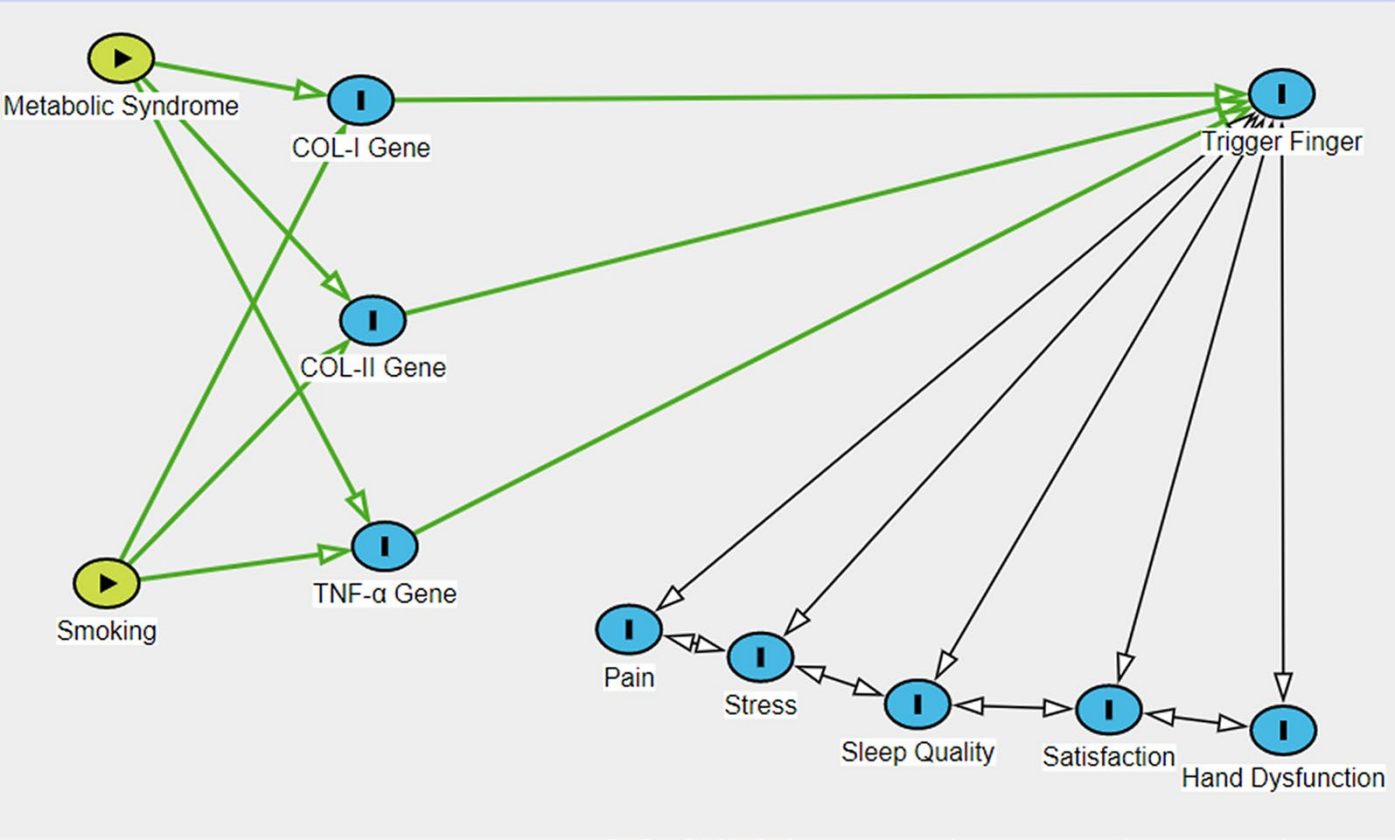
Directed acyclic graph (DAG) demonstrating the relationship between metabolic syndrome, smoking, gene expression and health-related factors

Table 5 shows the multivariate comparisons between groups. The mean value for COL-I, COL-II and TNF-α genes’ expression level was higher in the trigger finger groups. The mean score COL-I, COL-II and TNF-α genes’ expression level was affected by sleep, stress, hand function and pain, which served as confounding factors, although COL-I was not affected by sleep and stress and COL-II also was not affected by stress (Fig. 2).

**Table 5:** Multivariate analysis between groups (adjusted for the potential confounding effects of sleep, stress, hand function and pain)

Variables	Total (*n* = 45)	Healthy Control (*n* = 10)	Trigger Finger Five Groups (*n* = 35)	Group Effect *p*-Value	Sleep *p*-Value	Stress *p*-Value	Hand-Function *p*-Value	Pain *p*-Value	Satisfaction *p*-Value	Sleep-, Stress-, Hand-Function-, Pain and Satisfaction *p*-Value
COL-I gene expression level	1.925 (0.672)	1.241 (0.071)	2.142 (0.081)	0.0001 *	0.077	0.126	0.008 *	0.03 *	0.011 *	0.012 *
COL-II gene expression level	1.631 (0.272)	1.224 (0.177)	1.727 (0.097)	0.0001 *	0.001 *	0.826	0.0001 *	0.0001 *	0.0001 *	0.006 *
TNF-α gene expression level	2.461 (0.975)	1.260 (0.152)	2.711 (0.013)	0.0001 *	0.0001 *	0.0025 *	0.0001 *	0.0001 *	0.0001 *	0.0001 *

^*^P < *0.05*

### Differences in hand function between TF, TF with metabolic syndrome diseases and smoker TF patients

**Fig. 3:**
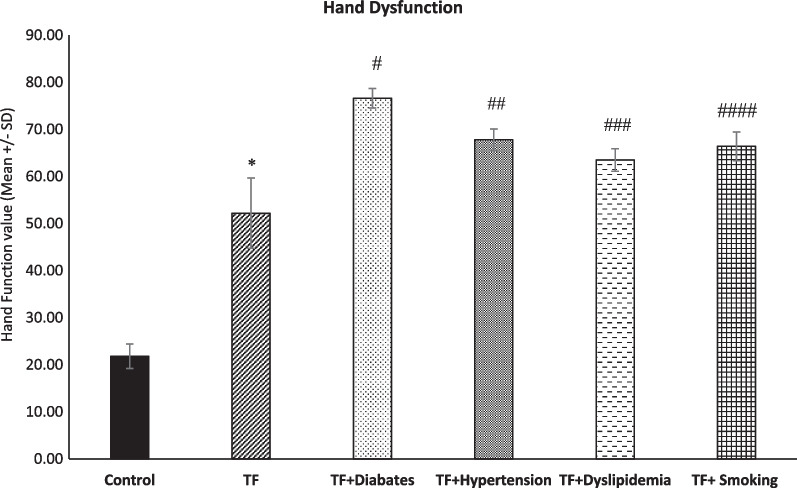
Level of hand dysfunction among groups (*n* = 45, mean + / − SE). Hand dysfunction was significantly higher among the TF groups in comparison with the control group (*p < 0.0001). The other experimental groups’ hand dysfunction significantly increased in comparison with the TF groups (^#, ##, ###, ####^*p* > 0.0001)

A statistically significant large effect of TF was found for DASH assessment compared with healthy control (*p* < 0.0001), as shown in Fig. 1. Post hoc analysis revealed statistically significant differences between TF with metabolic syndrome, smoker trigger finger patients and TF regarding hand function (^#^, ^##^, ^###^, ^####^ *p* > 0.0001, respectively), as shown in Fig. 3.

### Differences in stress level between TF, TF with metabolic syndrome diseases and smoker TF patents

**Fig. 4:**
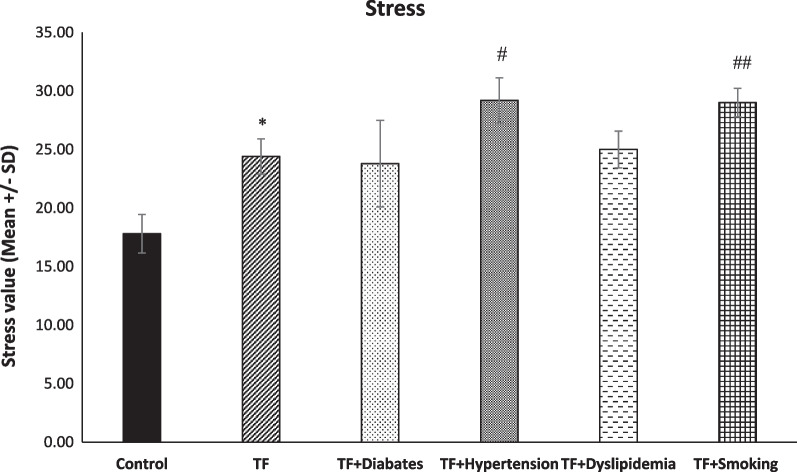
Level of stress among groups (*n* = 45, mean + / − SE). Stress was significantly increased in the TF group in comparison with the control group (*p < 0.001). When compared to the TF group, stress significantly increased in the TF + hypertension group (^#^*p* > 0.02). Additionally, stress was significantly increased among the TF + smoking groups in comparison with the TF group (^##^*p* > 0.031)

Regarding stress level, there was a significant difference between the control group and TF group (* *p* < 0.001). Moreover, the analyses showed that stress level was significantly higher in the TF patients with hypertension diseases and smoker TF patients compared to TF patients, with (^#^ *p* > 0.02) and (^##^ *p* > 0.031), respectively (see Fig. 4).

### Differences in sleep quality between TF and TF with metabolic syndrome diseases and smoker TF patients

**Fig. 5:**
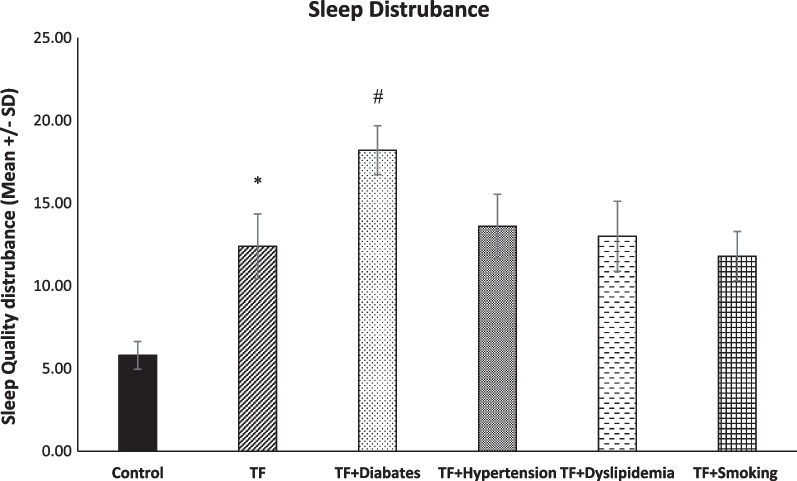
Level of sleep disturbance among groups (*n* = 45, mean + / − SE). A significant disturbance of sleep was evident in the TF groups in comparison with the control group (*p < 0.0001). Comparing the TF group to the TF + diabetes group, it was evident that the level of sleep disruption in the diabetic group was significantly higher (^#^*p* > 0.0001)

A significant difference between the TF and control (* p < 0.0001) groups is demonstrated in Fig. 5. However, there was no significant difference between TF and smoker TF, TF with dyslipidaemia, or TF with hypertension (*p* < 0.05). The diabetic TF group showed a significant disturbance in sleep quality compared to the TF group (^*#*^* p* < 0.0001) (Fig. 5).

### Differences in pain and life satisfaction between TF and TF with metabolic syndrome diseases

**Fig. 6:**
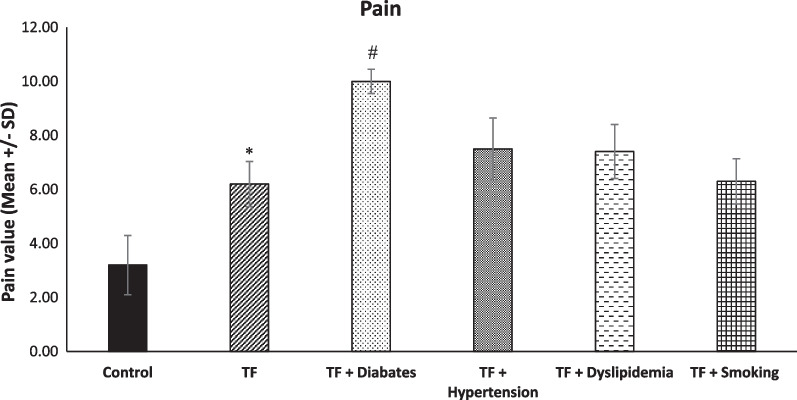
Level of pain among groups (*n* = 45, mean + / − SE). A significant increase in levels of pain was evident in the TF group in comparison with the control group (*p < 0.0001). The pain level in the TF + diabetes group was significantly greater in comparison with the other TF groups (^#^*p* > 0.0001)

**Fig. 7:**
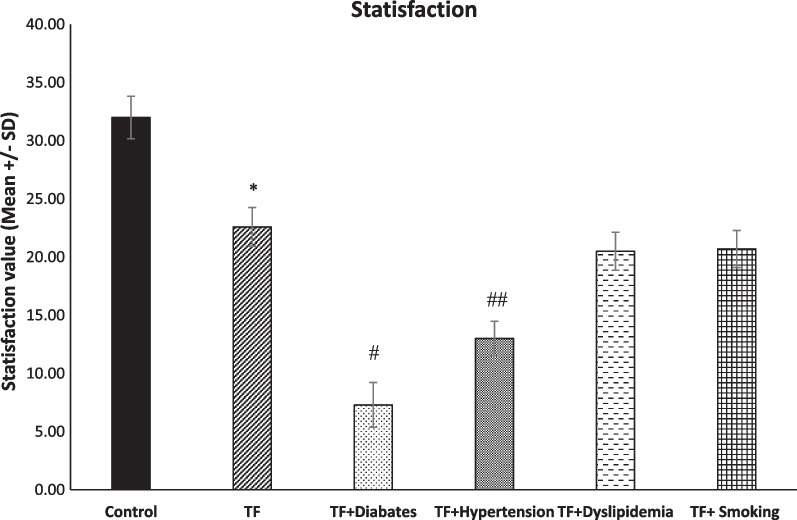
Level of life satisfaction among groups (*n* = 45, mean + / − SE). All the TF groups had significantly lower levels of life satisfaction in comparison with the control group (*p < 0.011). The life satisfaction level in the TF + diabetes and TF + hypertension group was significantly lower in comparison the TF group (^#, ##^*p* > 0.0001)

Regarding pain, there was a significant difference between control and TF groups (* *p* < 0.0001). In addition, the analyses showed that pain was significantly higher in the diabetic TF group in comparison with other TF groups (^*#*^* p* < 0.0001) (Fig. 6). Similarly, life satisfaction was also significantly lower in the diabetic and hypertension TF groups in comparison with the TF group (*p < 0.0001) (Fig. 7).

### Carpel tunnel syndrome (CT) versus trigger finger (TF)

**Fig. 8:**
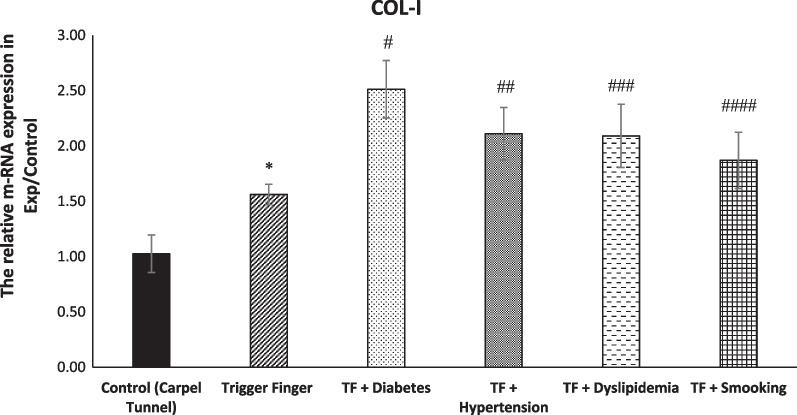
Level of mRNA expression of COL-I among groups (*n* = 43, mean + / − SE). A significant increase in COL-I expression was evident in the TF groups in comparison with the control group (*p < 0.0001). Comparing the TF group to the other experimental groups showed a significant increase in the mRNA expression of COL-I (^#,^^##,^^###,^^####^*p* > 0.0001)

**Fig. 9:**
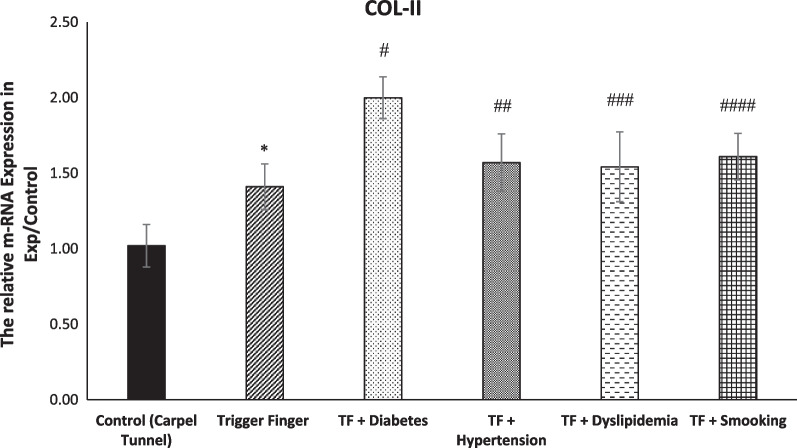
Level of mRNA expression of COL-II among groups (*n* = 43, mean + / − SE). COL-II was significantly increased in the TF group in comparison with the control group (*p < 0.05). The other experimental groups’ COL-II expression was significantly higher in comparison with the TF group (^#^*p* > 0.0001)

**Fig. 10:**
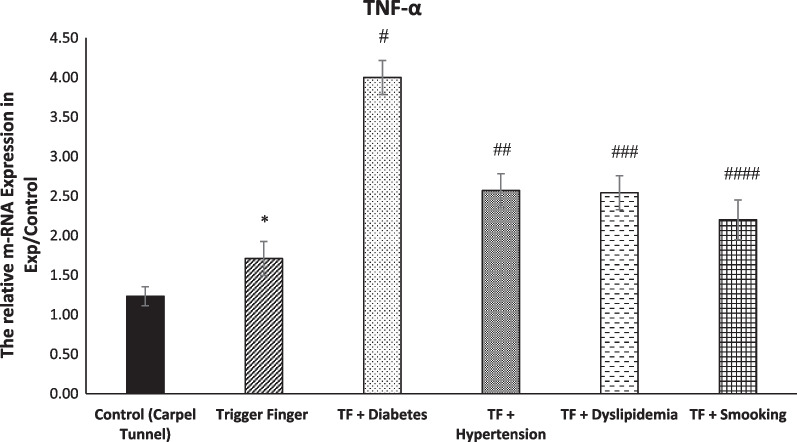
Graph comparing the mRNA expression of TNF-α of all TF groups (*n* = 43, mean + / − SE). TNF-α was significantly higher in the A1 pulley of TF group comparing to control group (*p > 0.0001). Moreover, the mRNA expression of TNF-α in the pulley A1 of experimental groups was significantly higher than TF group (^#, ##, ###, ####^*p* > 0.0001)

The COL-1, COL-2, TNF-α mRNA expression in trigger fingers was significantly higher when compared to samples from carpel tunnel syndrome (Figs. 8, 9, 10).

### trigger finger versus metabolic (TF) groups and smoking (TF) group

The COL-1, COL-2, TNF-α mRNA expression in trigger fingers with diabetes, hypertension and dyslipidaemia was significantly increased compared to the TF group. Moreover, The COL-1, COL-2, TNF-α mRNA expression in smoking trigger fingers in the A1 pulley was significantly higher in comparison with the non-smoker TF group (Figs. 8, 9, 10).

### TNF-alpha immunoblotting

**Fig. 11:**
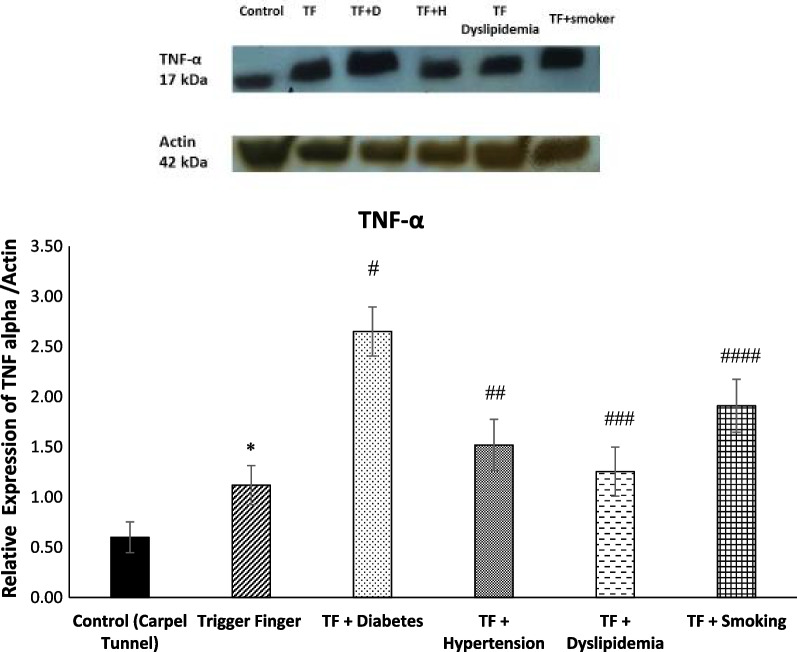
Western immunoblot protein analysis of TNF-alpha in the A1 pulley system of the 5 TF groups toward the conclusion of the experiments (*n* = 43, mean + / − SE). Densitometry analysis of TNF-α showed significantly higher TNF-α in the A1 pulley of TF group compared to control group (*p > 0.0001). Moreover, the expression of TNF-α in the pulley A1 of experimental groups was significantly higher than TF group (^#, ##, ###, ####^*p* > 0.0001)

The analysis for the TNF-alpha protein densitometry (band size is 17 kDa) are presented in Fig. 11. The diabetic TF group had significantly higher TNF-α protein expression in comparison with the control as well as the other TF groups (*p* < 0.0001).

There was correlation COL-1, COL-2, and TNF- α mRNA expression with hand dysfunction, sleep disturbance, and pain. Expect TNF- α and stress were there was no correlation as it shown in (Table 6).

**Table 6:** Pearson correlation between variables

	Hand dysfunction	Stress	Sleep disturbance	Pain	Satisfaction
*COLI*					
	.981**	.641**	.897**	.891**	.892**
	.000	.002	.000	.000	.000
N	45	45	45	45	45
*COLII*					
	.842**	.531**	.791**	.799**	.851**
	.000	.004	.000	.000	.000
N	45	45	45	45	45
*TNF*					
	.821**	.422	.815**	.847**	.881**
	.000	.061	.000	.000	.000
N	45	45	45	45	45

^*^Correlation is significant at 0.05

^**^Correlation is significant at 0.01

## Discussion

### Pain in metabolic syndrome trigger finger patients and smoker trigger finger patients

In this study, the pain level was evaluated in patients before undergoing surgical release of the A1 pulley using the numeric pain scale. According to one study conducted in 2020, diabetics have much greater prevalence of severe symptoms such as pain [23]. As shown in our results section, diabetic patients who developed trigger finger scored the highest among the groups, which is interpreted as severe pain. Indeed, trigger finger pain was evident in all the groups. Nonetheless, in some recent literature, hyperglycaemia was hypothesized to create a cross-link between collagen molecules, leading to collagen buildup in the tendon sheaths around the flexor tendon, which does not occur in non-diabetic trigger finger [24]. This process seems to be the reason why trigger finger pain is worse in diabetics. This correlation between diabetes and trigger finger was first suspected and confirmed in 1977 by Storm, then by Benedetti at 1982 and Yosipovitch, who suggested that a substantial and paramount factor in the pathogenesis of diabetic hand syndrome was the abnormal collagen metabolism, and that this was responsible for the stenosis seen in trigger finger and resulting proliferation of fibrous tissue [25].

Although it is postulated that nicotine was related to a higher incidence of trigger finger, when it comes to the severity of pain, smoker trigger finger patients were shown the same level of pain as non-smoker trigger finger patients [26]. To our knowledge, there is no existing research investigating the relationship between smoking and trigger finger. This study evaluated pain in smoker trigger finger using a sensitive, reliable, abs valid measure as the numerical pain scale. Despite of numerous studies highlighted that nicotine causes permanent damage to the hands [27], the surprising fact found in this study is that both trigger finger patients and smoker trigger finger patients have approximately the same score regarding the severity of pain.

Regarding the relationship between trigger finger and dyslipidaemia, there is no direct evidence to suggest a causal relationship between dyslipidaemia and trigger finger. However, one study claimed that high cholesterol levels can contribute to the development of conditions such as atherosclerosis, which can lead to reduced blood flow to the tendons and other tissue [28]. The reason for the extreme pain found in some patients with trigger finger and dyslipidaemia is probably a process caused by xanthoma, which is a known condition that occurs when high cholesterol levels lead to the formation of deposits in the tendon, which eventually causes excessive pain. Dyslipidaemia is a significant contributor to the development of diabetic neuropathy via inducing oxidative stress in root ganglia sensory neurons [29]. This is in linked with our results.

In the results section, our findings suggest that the presence of hypertension in patients, along with trigger finger, escalate the pain level. Previous studies found that hypertension can lead to inflammation and the deposition of collagen in the tendons, which may lead to pain [30].

### Hand dysfunction in metabolic syndrome trigger finger patients and smoker trigger finger patients

The significant difference between a person with trigger finger and a person with trigger finger and diabetes is noticeable when using the DASH assessment. Diabetic patients may struggle to perform functional tasks due to impaired sensory functioning, such as touch, pain, temperature, and proprioception, in their upper extremities, particularly their hands [31]. An article by Casanova et al. [32] describes three assessments that demonstrated decreased hand function in diabetic patients, which supports our results.

Several studies concluded that smoking negatively influences the healing process in general and deteriorates multiple medical conditions [33, 34]. Surprisingly, the smoking + TF group obtained an inconsequential DASH score compared to other groups, excluding the TF group. Similar to this, Samona et al. [35] claimed that despite the known negative consequences of smoking, their research did not support the proposed assertion that smoking has a substantial impact on the postoperative range of motion in flexor tendon injuries.

The trigger finger and dyslipidaemia may not necessarily go hand in hand, although dyslipidaemia scored the lowest in the DASH assessment when compared to other groups, with the exception of the TF group. According to a study measuring the connection between adult dyslipidaemia and hand grip strength (RGS), there was a negative correlation between relative HGS and the likelihood of dyslipidaemia [36].

There was an association between hypertension and decreased hand function among TF patients. Although this point was not addressed in previous studies, a study by Ji et al. [37] noted that a strong hand grip may be related to a higher risk of hypertension, which supports our results.

### Sleep disturbance in metabolic syndrome trigger finger patients and smoker trigger finger patients

After spending a lot of time with the hand in a fist while sleeping, “triggering” frequently occurs at night or in the morning. TF is more frequent in diabetics than in the general population, affecting the sleep pattern. According to a Swedish study performed by Lund University, having high blood sugar raises your likelihood of developing an issue. Using the Pittsburgh Sleep Quality Index (PSQI), our findings clearly demonstrated a significant difference between TF and TF + diabetes in terms of sleep patterns. Based on [38] smoking at night is substantially linked to increased insomnia and shorter sleep duration. Unexpectedly, the PSQI assessment revealed that there was no discernible difference between TF and TF + smoking in our data. The lowest result was found compared to other groups. The sleep patterns of a person with TF and a person with TF + smoking are approximately the same.

A short sleep duration and insomnia were strongly linked to the incidence of dyslipidaemia, although its link to poor sleep quality and the risk of obstructive sleep apnea was statistically insignificant [39]. This means that it could affect sleep patterns but not as much as other diagnoses. The PSQI results support the idea that there is no significant difference between TF and TF + dyslipidaemia.

In affluent societies, poor sleep is a growing problem that may contribute to adults' increased risk of hypertension. Comparing people who slept normally to those with higher PSQI scores, the likelihood of developing prevalent hypertension was considerably higher. According to Chen et al.'s [40] findings from 2022, people who had poor sleep quality had a 17% higher probability of having prevalent hypertension. This is consistent with our findings since, after TF + diabetes, TF + hypertension received the second highest score on the PSQI evaluation.

### Stress in in metabolic syndrome trigger finger patients and smoker trigger finger patients

In this study, the level of stress among our groups was presented, and TF + smoking groups had the highest score, followed by TF + hypertension. Few studies have been conducted related to TF + smoking. In addition, smokers frequently claim that quitting smoking reduces their stress levels. However, smokers experience slightly more stress than non-smokers [41, 42]. Regarding the TF + hypertension group, our bodies respond to stressful situations by releasing stress hormones (adrenaline and cortisol) into the blood, in addition to emotional discomfort. These hormones speed up heartbeat and tighten the blood vessels to direct more blood to the center of the body rather than the extremities as the body prepares for the “fight or flight” reaction. Blood pressure is briefly raised by blood vessel constriction and an increase in heart rate, but this effect only lasts until the stress reaction subsides. After that, blood pressure returns to its pre-stress level. Situational stress has symptoms that are typically transient and go away once the stressful event has passed. When we are confronted with an immediate threat that we can handle by facing or escaping, “fight or flight” is a useful response. However, there are many stressful events in the modern world that we are unable to manage using those solutions. Our bodies shift into high gear intermittently for days or weeks at a time while under chronic stress. Chronic stress and blood pressure have unclear relationships that are still being researched [43].

### Satisfaction with life in in metabolic syndrome trigger finger patients and smoker trigger finger patients

Life satisfaction is highly linked with better healthy lifestyle behaviors and healthier biologic functions. The results in our study showed a significant decrease in life satisfaction in the diabetic trigger finger group. This is well understood, as life satisfaction stems from harmony of one’s set of standards in life with life circumstances over longer timeframe [44]. Petterson et al. [45] conducted a study that included 1000 diabetic participants and showed that patients with a longer duration of diabetes exhibited lower levels of energy, and overall well-being, as well as higher levels of depression. Among the DM patients interviewed, 64% of participants expressed dissatisfaction with their life [46].

In our study, there was a significant decrease in life satisfaction among TF + hypertension group. A study showed that people in Stage 2 hypertension are less satisfied with their lives than people with lower blood pressure values [47]. Similarly, a cross sectional study reported that life satisfaction has a statistically significant negative correlation with blood pressure [48].

### COL-II, COL-II, and TNF-α genes and protein expression in in metabolic syndrome trigger finger patients and smoker trigger finger patients

Presenting of the first preliminary data of epigenetic changes in gene expression in metabolic syndrome trigger fingers and smoker trigger fingers is explored by our study. The development of pathological complications resulting from chronic inflammation is associated with the inappropriate or increased activation of TNF-α signaling [49]. Our results showed a significant increase in the expression of TNF-α among all the TF groups. The significant increase in the TF + metabolic syndrome groups was expected, as Sookoian et al. [50], found that TNF-α is associated with the pathological process of metabolic trigger fingers. Diabetes and insulin resistance are linked to a high level of TNF-α in the blood [49]. Obesity and type 2 diabetes are both associated with TNF-alpha [51]. The increased mRNA expression of TNF-α was expected in smokers’ TF tendons. Based on the results of Petrescu et al. [52], smokers' serum contains high amounts of TNF-, which may indicate an imbalance between pro- and anti-inflammatory factors due to tobacco smoke exposure.

Collagens such as COL-II AND COL-II and other protein structures form the ECM. Changes in ECM result in rapid alterations in the mechanical properties of ECM [53]. Our results showed mRNA expression to have significantly increased in the TF groups of both COL-I and COL-II. COL1A1 genes could be regarded as novel diagnostic biomarkers that predict the progression of human lung cancer [54]. Collagen and other extracellular matrix structures are more frequently produced in diabetes with chronic hyperglycaemia, and they are deposited in an irregular manner [55]. Similar to our results, a study conducted by Cain et al. [21], showed a significant increase in the mRNA expression of both COL-I and COL-II among diabetic trigger fingers because these tendons undergo epigenetic modifications as a result of chronic hyperglycaemia.

## Limitations and future directions

A small sample size and the few behavioral, clinical, and biochemical parameter measures are the study's main limitations. To come to definitive conclusions regarding the variation in health-related factors and genes related to epigenetics, future studies with a large sample size are required. In the future research, rate of analgesia should be evaluated clinically for users suffering from chronic pain. Moreover, the possible role of TF development other than epigenetic factors, polymorphism of some genes such as KLHL1, POLE2, could be investigated in the future researches.

## Conclusions and implications

The extent of hand dysfunction, stress level, and pain severity in the TF tendons was highly related to the degree of inflammation and genetic alterations in TF metabolic syndromes and smoker TF patients. Therefore, further rigorous research is needed to investigate the integration of health-related factors, gene expression and occupational therapy as a promising approach to the management of TF. A combination of occupational therapy and molecular genetics is a new promising approach to restoring normal function and reducing the risk of resistance to conservative therapy or recurrence. Moreover, an understanding of the molecular signaling pathways involved in the pathogenesis is needed to design therapeutic strategies for the treatment of trigger finger. The level of inflammation in each group determines which group has the best and worst prognosis.

## Data Availability

On reasonable request, the data can be obtained from the corresponding author.
